# Knock-in human *FGFR3* achondroplasia mutation as a mouse model for human skeletal dysplasia

**DOI:** 10.1038/srep43220

**Published:** 2017-02-23

**Authors:** Yi-Ching Lee, I-Wen Song, Ya-Ju Pai, Sheng-De Chen, Yuan-Tsong Chen

**Affiliations:** 1 Institute of Cellular and Organismic Biology, Academia Sinica, Taipei, 11529, Taiwan; 2 Institute of Biomedical Sciences, Academia Sinica, Taipei, 11529, Taiwan; 3 Department of Pediatrics, Duke University Medical Center, Durham, NC, 27710, USA

## Abstract

Achondroplasia (ACH), the most common genetic dwarfism in human, is caused by a gain-of function mutation in fibroblast growth factor receptor 3 (*FGFR3*). Currently, there is no effective treatment for ACH. The development of an appropriate human-relevant model is important for testing potential therapeutic interventions before human clinical trials. Here, we have generated an ACH mouse model in which the endogenous mouse *Fgfr3* gene was replaced with human *FGFR3*^*G380R*^ (*FGFR3*^*ACH*^) cDNA, the most common mutation in human ACH. Heterozygous (*FGFR3*^ACH/+^) and homozygous (*FGFR3*^ACH/ACH^) mice expressing human *FGFR3*^G380R^ recapitulate the phenotypes observed in ACH patients, including growth retardation, disproportionate shortening of the limbs, round head, mid-face hypoplasia at birth, and kyphosis progression during postnatal development. We also observed premature fusion of the cranial sutures and low bone density in newborn *FGFR3*^G380R^ mice. The severity of the disease phenotypes corresponds to the copy number of activated *FGFR3*^*G380R*^, and the phenotypes become more pronounced during postnatal skeletal development. This mouse model offers a tool for assessing potential therapeutic approaches for skeletal dysplasias related to over*-*activation of human *FGFR3*, and for further studies of the underlying molecular mechanisms.

Gain-of-function point mutations in fibroblast growth factor receptor 3 (*FGFR3*) cause a variety of congenital skeletal dysplasias inherited as an autosomal dominant trait. These skeletal dysplasias are characterised by varying degrees of skeletal deformities ranging from least to most severe as follows: hypochondroplasia (HCH), achondroplasia (ACH), severe achondroplasia with developmental delay and acanthosis nigricans, and thanatophoric dysplasia (TD) types 1 and 2. Achondroplasia (ACH) (OMIM 100800) is the most common form of genetic short-limbed dwarfism in human. ACH is characterised by short stature with disproportionately short limbs, macrocephaly, characteristic faces with frontal bossing, midface hypoplasia, and exaggerated thoracolumbar kyphosis^1^. Over 99% of individuals affected with ACH have the same point mutation, G380R, in the transmembrane domain of *FGFR3* protein (*FGFR3*^G380R^)^2^,^3^. The clinical features of heterozygous ACH are consistent among patients, and homozygous ACH causes severe skeletal deformities that lead to early death. The majority of ACH cases (over 80%) occur spontaneously through mutations in sperm related to advanced paternal age^4^.

*FGFR3* is expressed mainly in proliferating chondrocytes in the developing long bones^5^ and has been proven to be a negative regulator of endochondral bone growth^6^. Morphometric examination revealed the shortening of growth plates in ACH patients^7^. Several ACH mouse models have been established to study the roles of *FGFR3* in skeletal development and disease. Three ACH mouse models express murine ACH mutation (*Fgfr3*^*ACH*^), introduced using knock-in^8^,^9^ or transgenic^10^ approaches, and one ACH mouse model transgenically expresses human *FGFR3*^ACH^ 11. These mouse models share some ACH phenotypes. However, some phenotypes have not been fully described or examined in these models.

Currently, there is no effective treatment for skeletal dysplasias caused by activating mutations of *FGFR3*. Several potential therapeutic strategies targeting either the over-activated *FGFR3* or its downstream effects are currently under development. An *FGFR3*-binding peptide^12^, a C-type natriuretic peptide analogue^13^, a soluble form of human *FGFR3*^14^, parathyroid hormone^15^, and statins^16^ have been shown to improve bone growth in genetically manipulated ACH or TD I mouse models *in vivo* or *ex vivo*. The effects of statins have been examined using the *in vitro* human-relevant model of chondrocytes differentiated from induced pluripotent stem cells from either TD I or ACH patients. The C-type natriuretic peptide analogue has reached clinical trials^17^. It is important to develop a human-relevant *in vivo* model to provide a robust system for testing potential therapeutic interventions before human clinical trials.

In this report, we developed a human-relevant ACH mouse model by replacing mouse *Fgfr3* with human *FGFR3* cDNA containing the *FGFR3*^G380R^ ACH mutation. The clinical phenotypes and histology of bone abnormalities were characterised in the mutant mice. This *FGFR3*^*ACH*^ mouse model closely recapitulates human ACH. As such, it offers a valuable tool for assessing potential therapeutic approaches designed to target the over-activation of human *FGFR3*.

## Results

### Generation of *FGFR3* ^*ACH*^ and *FGFR3* ^*WT*^ mice

**Figure 1:**
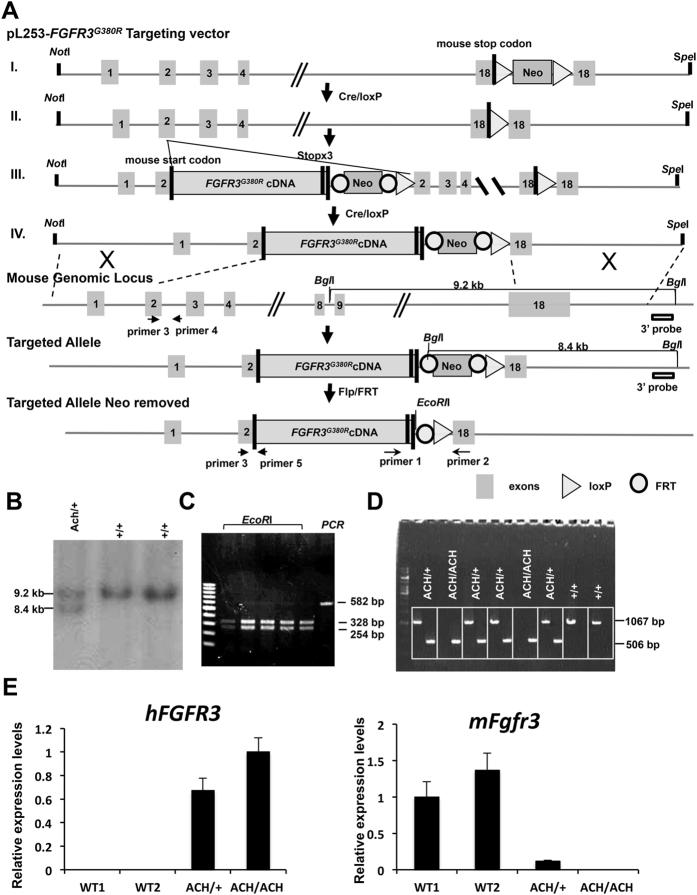
Generation of ACH mice and human *FGFR3* WT controls by introducing human *FGFR3*^*G380R*^ cDNA or WT *FGFR3* into the murine *Fgfr3* locus. (**A**) Strategy for the generation of the targeting vector and depiction of the final chromosomal structure of the murine *Fgfr3* locus after the introduction of the human *FGFR3*^*G380R*^ cDNA via gene targeting. (**B**) Mouse embryonic stem cell clones containing the targeted allele were identified by Southern blot analysis. (**C**) The neomycin resistance cassette in the identified stem cells was removed by Flp/*FRT* excision and analysed by PCR amplification and EcoRI digestion. A 528 bp PCR product was present in the stem cells without the neomycin resistance cassette, and the 328 bp and 254 bp fragments produced by EcoRI digestion of the PCR product could be detected. (**D**) PCR amplification analysis of genomic DNA isolated from WT and *FGFR3*^*ACH*^ mice. A 1067 bp PCR product was amplified from the mouse *Fgfr3* locus. A 506 bp PCR product was amplified from the human *FGFR3*^G380R^ targeted allele. (**E**) The mRNA expression of targeted human *FGFR3*^G380R^ and endogenous mouse *Fgfr3* in the heterozygous *FGFR3*^G380R^, homozygous *FGFR3*^G380R^, and WT mice was determined by RT-PCR using sequence-specific primers. ACH/+, the heterozygous *FGFR3*^*ACH*/+^ mice; ACH/ACH, the homozygous *FGFR3*^*ACH/ACH*^ mice; +/+, wild type littermates.

To generate *FGFR3*^*ACH*^ mice, we used a gene-targeting approach to replace the mouse *Fgfr3* with human *FGFR3* cDNA carrying the ACH mutation (*FGFR3*^*ACH*^) under the full control of the endogenous mouse *Fgfr3* promoter, intron 1, and 5′ and 3′ untranslated regions (Fig. 1A). Human WT *FGFR3 (FGFR3*^*WT*^) cDNA was introduced into *Fgfr3* through the same approach to generate control mice for comparison. Southern blotting (Fig. 1B) and polymerase chain reaction (PCR) of genomic DNA detected the *FGFR3*^*ACH*^ cDNA within *Fgfr3* in embryonic stem cells (Fig. 1C). PCR of genomic DNA detected the human *FGFR3*^*ACH*^ cDNA and mouse genomic *Fgfr3* DNA from heterozygous (*FGFR3*^*ACH*/+^), homozygous (*FGFR3*^*ACH/ACH*^) and WT mice (Fig. 1D). The expression of the human *FGFR3*^*ACH*^gene and endogenous mouse *Fgfr3* gene in ACH and WT mice was determined in the left hind-limb of neonatal mice by RT-PCR using gene specific primers (Fig. 1E).

### Skeletal abnormalities in newborn *FGFR3* ^ACH^ mice

**Figure 2:**
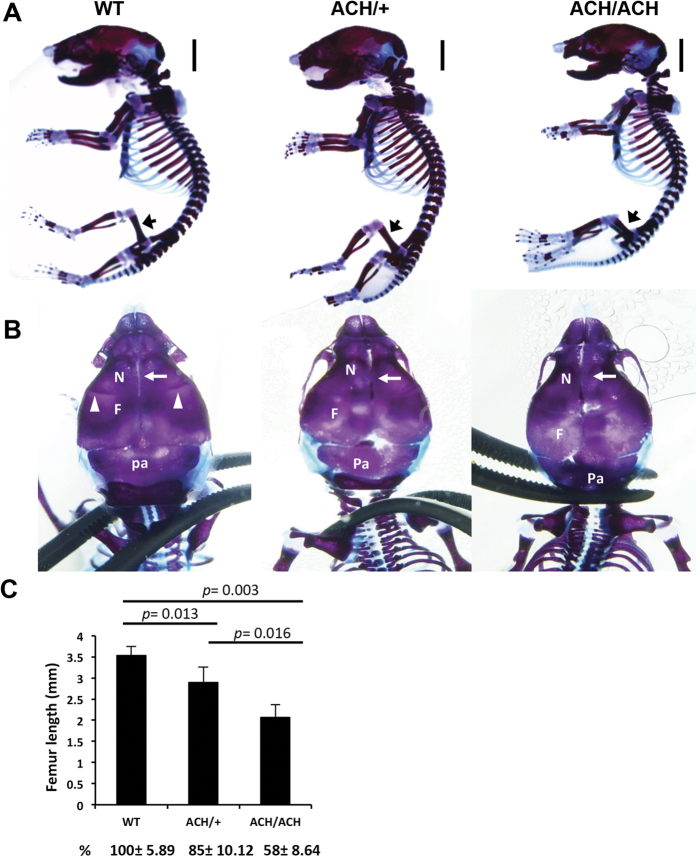
Skeletal defects and changes in bone architecture in newborn *FGFR3*^*ACH*^ mice. (**A**) Lateral view of skeletal preparations of *FGFR3*^*ACH*/+^ (ACH/+), *FGFR3*^*ACH/ACH*^ (ACH/ACH), and WT mice. Cartilage was stained with Alcian blue and bone was stained with alizarin red. Scale bar: 30 mm. (**B**) Dorsal view for comparison of the skulls. The white arrowheads indicate the jugum limitans, and the white arrow indicates the metopic suture. N, nasal bones; F, frontal bones; Pa, parietal bones. (**C**) Femur length was significantly decreased in *FGFR3*^*ACH/ACH*^ and *FGFR3*^*ACH*/+^ mice. WT, *n* = 3; ACH/ + , *n* = 6; ACH/ACH, *n* = 3.

The features of human ACH patients can be readily identified clinically and radiologically at birth. At birth, there were no obvious differences in appearance between *FGFR3*^*ACH*/+^ or *FGFR3*^*ACH/ACH*^ mice, collectively termed *FGFR3*^*ACH*^, and their WT littermates (Supplemental Fig. 1A). We therefore analysed the bone structure of newborn mice. The newborn *FGFR3*^*ACH*^ mice showed proximal limb shortening with relatively normally sized trunks (Fig. 2A). Femur length was reduced by 15% in *FGFR3*^*ACH*/+^ mice and 42% in *FGFR3*^*ACH/ACH*^ mice compared with WT mice (Fig. 2C). A closer view of the skull structure revealed the skull was rounded and the calvarial bones were distorted in *FGFR3*^*ACH*^ mice, due to a positional shift and compression of the frontal and parietal bones (Fig. 2B). The jugum limitans, i.e., the cranial suture that separates the frontal and nasal bones, was absent in *FGFR3*^*ACH*^ mice (Fig. 2B). The metopic sutures, which line the midline between the two nasal bones, were unilaterally fused or partially absent in *FGFR3*^*ACH*^ mice (Fig. 2B). Thus, newborn *FGFR3*^*ACH*^ mice exhibited premature suture closure and abnormal skull shapes. Furthermore, a shorter intervertebral distance between cervical vertebrae (Supplemental Fig. 1B) and a narrower rib cage (Supplemental Fig. 1D) were observed in *FGFR3*^*ACH*^ newborns. These phenotypes are similar in many respects to the skeletal deformities in human ACH newborns^18^, and the bone abnormalities are more evident in *FGFR3*^*ACH/ACH*^ mice than in *FGFR3*^*ACH*/+^ mice.

### Pronounced skeletal abnormalities in *FGFR3* ^ACH^ mice during postnatal development

**Figure 3:**
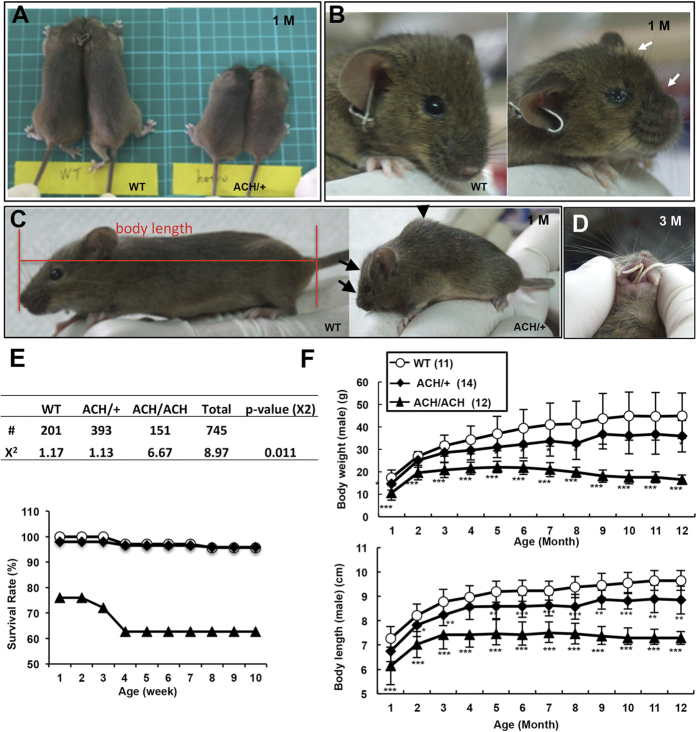
The appearance, survival rates, and growth kinetics of *FGFR3*^*ACH*^ and WT mice. (**A**) The ACH phenotypes observed in 1-month-old *FGFR3*^*ACH*/+^ mice (ACH/ + ) (right panel) as compared with the WT littermates (WT) (left panel). Dwarfism in *FGFR3*^*ACH*/+^ mice presented as a rounded head and a short snout, also indicated by white arrows in (**B**) and black arrows in (**C**). (**C**) Severe kyphosis (humpback), as indicated by the black arrowhead. (**D**) Protruding incisors presented in 3-month-old *FGFR3*^*ACH*/+^ mice. (**E**) Survival rates at birth (upper panel) and survival curves (lower panel) of the *FGFR3*^*ACH*/+^, *FGFR3*^*ACH/ACH*^, and WT mice. #, The survival mice number at birth. χ^2^, Chi-square goodness-of-fit tests (2 degrees of freedom). *P* < 0.05 suggested deviations from Mendelian expectations. (**F**) Growth curves of male *FGFR3*^*ACH*/+^, *FGFR3*^*ACH/ACH*^, and WT mice. The body length, measured from nose to tail base as indicated in (**C**). Body weights and body lengths of mice were measured monthly from birth until 12 months of age. Each curve shows the average Body weights and body lengths of animals from several litters. Data points represent means ± SD; *n* values are shown in parentheses after group names.

The dwarfism phenotypes gradually became evident in *FGFR3*^*ACH*^ mice. Dominant short stature (Fig. 3A), rounded head (Fig. 3B,D), short snout (Fig. 3B,C), and kyphosis (humpback) (Fig. 3C) phenotypes could be readily observed in *FGFR3*^*ACH*^ mice at 10 days to 1 month of age. All *FGFR3*^*ACH*/*ACH*^ mice developed kyphosis phenotypes at around 2 weeks of age, and about 90% of *FGFR3*^*ACH*/+^ mice developed kyphosis phenotypes before 1 month of age. In addition, protrusion of the lower incisors was observed in *FGFR3*^*ACH*^ mice (Fig. 3D) because of changes in the skull affecting the alignment of the incisors. *FGFR3*^*ACH*/*ACH*^ mice had a significantly lower survival rate at birth relative to expectations and a higher mortality rate before 4 weeks of age compared with *FGFR3*^*ACH*/+^ and WT mice (Fig. 3E), and the majority of *FGFR3*^*ACH*^ mice died at around 1 year of age. Mean body weights and body lengths were decreased in *FGFR3*^*ACH*/+^ and *FGFR3*^*ACH/ACH*^ mice (Fig. 3F). *FGFR3*^*ACH*/+^ mice exhibited intermediate body weights and lengths between those of the WT and *FGFR3*^*ACH/ACH*^ mice, indicating a dose-dependent effect of activated *FGFR3*^G380R^. In contrast, the control *FGFR3*^*WT*/+^ or *FGFR3*^*WT/WT*^ mice expressing non-mutated human *FGFR3* showed identical external phenotypes to those of WT (Supplemental Fig. 2A). The growth rates of WT, *FGFR3*^*WT*/+^, and *FGFR3*^*WT/WT*^ mice were the same (Supplemental Fig. 2B).

**Figure 4:**
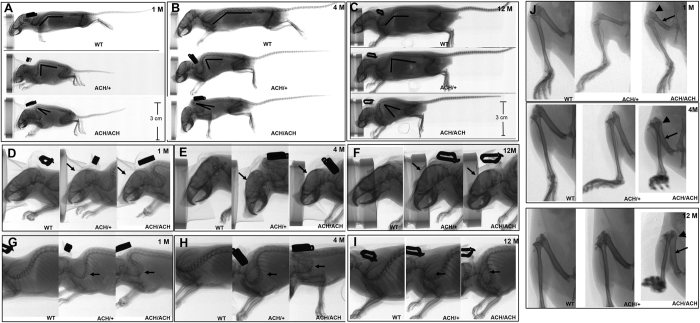
Radiographs of *FGFR3*^*ACH*/+^ (ACH/ + ), *FGFR3*ACH/ACH (ACH/ACH), and WT mice (WT). *FGFR3*^ACH^ mice showed profound rhizomelic dwarfism, a rounded skull, and kyphosis (arrow) in the cervico-thoracic spine. The severity of these phenotypes correlated with the dose of *FGFR3*^*ACH*^ and postnatal development. Lateral view of whole skeleton at the age of 1 month (**A**), 4 months (**B**), and 1 year (**C**). The enlarged view shows rounded skull at the age of 1 month (**D**), 4 months (**E**), and 1 year (**F**). The enlarged view shows kyphosis (arrow) in the cervico-thoracic spine in *FGFR3*^*ACH*/+^ and *FGFR3*^*ACH/ACH*^ mice aged 1 month (**G**), 4 months (**H**), and 1 year (**I**). The enlarged view shows short, curved, and thick bones with widened diaphyses and flared metaphyses in the femurs of *FGFR3*^*ACH*^ mice at several developmental stages (**J**).

Two-dimensional micro-computed tomography (micro-CT) was used to examine the skeletal abnormalities in *FGFR3*^*ACH*^ mice. The skeletal bone revealed dwarfism, rounded skulls, and severe curvature of the cervical and upper thoracic vertebrae in *FGFR3*^*ACH*^ mice (Fig. 4A–C). *FGFR*^*ACH/ACH*^ mice exhibited more severe phenotypes compared with those of *FGFR3*^*ACH*/+^ mice (Fig. 4A–C). Furthermore, these phenotypes became more pronounced in older mice (based on comparison among the phenotypes of 1-, 4-, and 12-month-old mice in Fig. 4A–C. Close observation of the skulls and vertebrae of *FGFR3*^*ACH*^ mice revealed shortened snouts and dome-shaped skulls (Fig. 4D–F), and almost completely folded upper thoracic vertebrae in *FGFR*^*ACH/ACH*^ and older *FGFR*^*ACH*/+^ mice (Fig. 4G–I). The severities of these phenotypes were more consistent among *FGFR3*^*ACH/ACH*^ mice, as compared with *FGFR3*^*ACH*/+^ mice, as shown by the smaller variation in the body lengths of *FGFR3*^*ACH/ACH*^ mice compared with that of *FGFR3*^*ACH*/+^ mice (Fig. 3F). This is relevant because the variation in the severities of the short snout, rounded-head, and kyphosis phenotypes is represented in the body length.

Patients with ACH present with rhizomelic (short-limbed) dwarfism. This phenotype was reproduced in the *FGFR3*^*ACH*/+^ mice, which showed a 22% shortening of femur length along with a 7.1% shortening of body length at 1 month of age, compared with the corresponding measurements in WT mice (Table 1). The results suggested that the limbs were disproportionately shortened relative to body length in *FGFR3*^*ACH/+*^ mice. Furthermore, the femurs were short, curved, and thick with widened diaphyses and flared metaphyses in *FGFR3*^*ACH*^ mice (Fig. 4J), which are very similar to phenotypes observed in ACH patients.

### Altered chondrocyte proliferation and differentiation in *FGFR3* ^*ACH*^ mice

**Figure 5:**
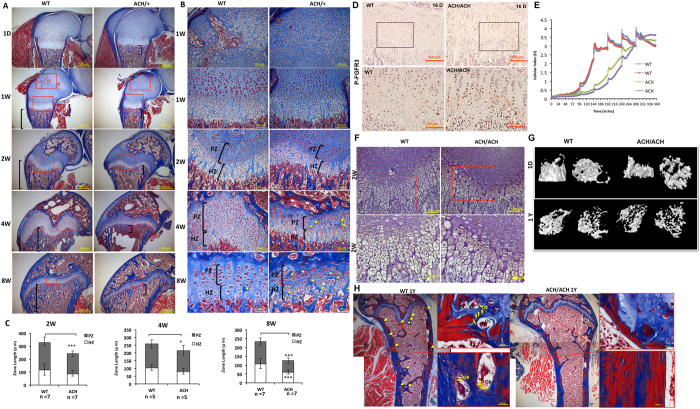
Histological, immunohistochemical, and micro-computed tomography analysis of distal femoral growth plates and chondrocyte proliferation in *FGFR3*^*ACH*/+^ (ACH/+), *FGFR3*ACH/^*ACH*^(ACH/ACH) and WT mice. The insets show magnified views. (**A**) Masson’s trichrome staining of distal femoral growth plates at the ages of 1 day, 1 week, 2 weeks, 4 weeks, and 8 weeks. Square brackets: the region of trabecular bone. Scale bar, 500 μm. The insets show magnified views in (**B**). Femoral growth plates show the typical zonal structure of proliferating chondrocytes (PZ) and hypertrophic chondrocytes (HZ) in *FGFR3*^*ACH*/+^ and WT mice. Delayed formation of secondary ossification centres was apparent in *FGFR3*^*ACH*/+^ mice at 1 week of age. Growth plates were shorter with more compacted proliferative cells in *FGFR3*^*ACH*/+^ mice. An increased amount of space (yellow arrows) between the chondrocyte columns can be appreciated in *FGFR3*^*ACH*/+^ mice at 4 and 8 weeks of age. (**C**) Quantitative measurements of the heights of the PZ and HZ of the distal femoral growth plates of *FGFR3*^*ACH*^ (ACH) mice and WT mice at 2, 4, and 8 weeks of age. Data represent mean values ± SD. *p < 0.05; ***p < 0.001. (**D**) Immunohistochemistry staining of phospho-*FGFR3* in growth plate chondrocytes showed increased abundance of phospho-*FGFR3* in 16-day-old *FGFR3*^*ACH/ACH*^. (**E**) An increased growth rate of chondrocytes from *FGFR3*^*ACH/ACH*^ analysed using an iCELLigence™ real-time cell analysis system. The data are presented as changes in the cell index over time. (**F**) Immunohistochemical analysis of osteocalcin showed increased abundance of osteocalcin in the chondrocytes of the hypertrophic zone in 2-week-old *FGFR3*^*ACH/ACH*^. (**G**) Vertical (left) and transverse (right) views of trabecular bone in the distal femoral metaphysis of WT and *FGFR3*^*ACH/ACH*^mice at 1 day and 1 year of age assessed using micro-computed tomography analysis. (**H**) Masson’s trichrome staining of the distal femurs of WT and *FGFR3*^*ACH/ACH*^mice at 1 year of age. The yellow arrows indicate the osteoblasts and osteoclasts. The insets show examples of the magnified views of osteoblasts (OB) and osteoclasts (OC).

Femur length is significantly reduced in *FGFR3*^*ACH*^ mice (Fig. 2C and Table 1). To examine defects in the long bones of *FGFR3*^*ACH*^ mice more closely, we performed a histological analysis of the distal femur from WT and *FGFR3*^*ACH*^ mice at different developmental stages. The epiphyseal structure was similar between the WT *FGFR3*^*ACH*^ mice at birth (Fig. 5A). The secondary ossification centre was readily formed in WT mice at 1 week of age, whereas its formation was markedly delayed in *FGFR3*^*ACH*^ mice (Fig. 5A,B), suggesting a delay in chondrocyte terminal differentiation. In endochondral ossification, chondrocytes sequentially transit through resting, proliferating, and hypertrophic stages. The *FGFR3*^*ACH*^ mice showed good development of each stage. However, the growth plates were significantly shorter in *FGFR3*^*ACH*^ mice with a shorter proliferative zone at 2, 4, and 8 weeks of age (Fig. 5B,C). This was caused by a reduction in the number of proliferative chondrocytes, indicating that chondrocyte proliferation was compromised in *FGFR3*^*ACH*^ mice. Despite the shorter proliferative zone, the arrangement of chondrocyte columns in the growth plate remained normal in *FGFR3*^*ACH*^ mice before 2 weeks of age. The disturbed arrangement of chondrocyte columns in *FGFR3*^*ACH*^ mice can be appreciated at 4 and 8 weeks of age, and their arrangement was disrupted by an increased amount of space between the columns (Fig. 5B). We further showed that *FGFR3*^*ACH*^ mice had higher *FGFR3* phosphorylation in chondrocytes of growth plates (Fig. 5D) and the primary chondrocytes had lower proliferation rates compared with those from WT mice (Fig. 5E), suggesting that *FGFR3* activation inhibited chondrocyte proliferation in *FGFR3*^*ACH*^ mice.

### Altered bone formation in *FGFR3* ^*ACH*^ mice

Low bone density has been reported in adult ACH patients^19^, which may have clinical relevance and lead to subsequent bone damage. The development of the long bones is coordinated between chondrogenesis and osteogenesis. Reduced growth of the longitudinal trabecular bone was observed in the distal femoral metaphysis of *FGFR3*^*ACH*^ mice at several stages of postnatal development (Fig. 5A, stained in blue). Furthermore, the expression of osteocalcin, which is associated with the early stages of matrix ossification, was increased in the chondrocytes of the hypertrophic zone of the distal femur of *FGFR3*^*ACH*^ mice at 2 weeks of age (Fig. 5F). A reduced hypertrophic zone was observed in *FGFR3*^*ACH*^ mice at 8 weeks of age (Fig. 5B,C). These results indicate that the bone-forming process was disturbed in *FGFR3*^*ACH*^ mice. To determine the structure of trabecular bone, we performed a micro-CT analysis. Three-dimensional images of the distal femoral metaphysis showed a lower bone volume with thinner and fewer trabecular bones and larger intertrabecular spaces in newborn and 1-year-old *FGFR3*^*ACH*^ mice compared to WT mice (Fig. 5G). A histomorphometric analysis of bone formation showed that the trabecular bone volume (BV/TV), trabecular thickness (Tb.Th), and trabecular number (Tb.N) were decreased, along with an increased trabecular separation (Tb.Sp) and structure model index (SMI) in the distal femoral metaphysis of *FGFR3*^*ACH*^ mice compared with WT mice at birth and at 1 year of age (Table 2). Furthermore, we observed fewer osteoblasts and osteoclasts in the femurs of *FGFR3*^*ACH*^ mice at 1 year of age (Fig. 5H), suggesting that the bone turnover rate might be altered in *FGFR3*^*ACH*^ mice.

## Discussion

In this study, we describe an ACH mouse model (*FGFR3*^*ACH*^), which expresses human *FGFR3*^G380R^, the most common mutation in human ACH patients. Our mouse model recapitulates the main human ACH phenotypes and offers a valuable tool for studying pathological conditions and testing potential therapeutic approaches designed to target the over-activation of human *FGFR3* and its downstream signaling pathway *in vivo*. In addition to the postnatal phenotypes observed in previous ACH mouse models^8^,^10^,^11^, we showed that *FGFR3*^*ACH*^ mice shared most of the key clinical phenotypes observed in human ACH patients at birth, including rhizomelic dwarfism, rounded skull, and midface hypoplasia. Furthermore, we described craniosynostosis and low bone density in newborn *FGFR3*^*ACH*^ mice. These phenotypes became more pronounced during postnatal skeletal development and were correlated with the dose of *FGFR3*^ACH^. Kyphosis, which is common in human ACH during postnatal development, develops postnatally in *FGFR3*^*ACH*^ mice. Although previously generated ACH mouse models share some ACH phenotypes, some phenotypes have not been fully observed or described in these ACH mouse models. Here, we described the full range of ACH abnormalities in this mouse model. A comparison of skeletal phenotypes between human achondroplasia and ACH mouse models is summarised in Table 3.

Previously, two ACH mouse models were generated by either targeting^8^ or transgenically expressing^10^ murine *Fgfr3*^*G374R*^(an ortholog of the human mutation *FGFR3*^*G380R*^, *Fgfr*^*ACH*^). Another ACH mouse model transgenically expresses human FGFR^G380R^ under the control of the mouse *Fgfr3* promoter region^11^. All these ACH mouse models exhibit some human ACH phenotypes during postnatal development. However, rhizomelic (short-limbed) dwarfism was not obvious, and dwarfism phenotypes were not described at birth in both *Fgfr*^*ACH*^ models. In addition, the transgenic model expressing *Fgfr3*^*ACH*^ under the control of the collagen II promoter presented some specific defects that are not usually observed in human ACH patients due to misexpression of *Fgfr3*^*ACH*^ 10. The transgenic human *FGFR3*^G380R^ mice presented the rhizomelic dwarf phenotype at birth^11^. However, thoracic kyphosis was not described for this model^11^. Moreover, homozygous *FGFR3*^G380R^ transgenic mice expressed more severe phenotypes than the two *Fgfr3*^*ACH*^ mouse models or our *FGFR3*^*ACH*^ model, and died shortly after birth. The severe phenotypes in homozygous *FGFR3*^G380R^ transgenic mice might be because the mice express both endogenous *Fgfr3* and human *FGFR3*^G380R^. Most homozygous ACH patients are stillborn or die during the neonatal period. Although our *FGFR3*^*ACH/ACH*^ mice exhibited a higher mortality rate than the WT mice at birth and before 4 weeks of age, 60% of *FGFR3*^*ACH/ACH*^ mice survived after 1 month in our study. The homozygous knock-in or transgenic *Fgfr3*^*ACH*^ mice survived after birth, but their neonatal survival rate was not reported. The human and mouse *FGFR3* genes share 92.7% identity and 94.9% similarity. The various phenotypes and different levels of severity observed in different ACH mouse models might be caused by the different promoter chosen and various copy numbers of activated *FGFR3* in the transgenic models, the different knock-in approaches used in the KI models, and the different genetic backgrounds of the mice assayed. Having access to various ACH mouse models provides the opportunity for choosing particular ACH phenotypes of interest for further studies. Nevertheless, the *FGFR3*^*ACH/ACH*^ mice generated in this study exhibited obvious and homogenous clinical phenotypes of ACH. Furthermore, these mice expressed human *FGFR3*^G380R^ alone without interference by endogenous *Fgfr3*, making them a useful model for further evaluating potential treatments targeting the over-activation of human *FGFR3* and its downstream signaling.

The biological basis of dwarfism phenotypes in ACH patients involves a specific defect in endochondral ossification and longitudinal bone growth^1^. The molecular consequences of over-activation of *FGFR3* on chondrocyte proliferation and differentiation in the growth plate have been well established^1^. Here, we demonstrated that over-activated human *FGFR3*^G380R^ has inhibitory effects on chondrocyte proliferation and maturation in an *in vivo* mouse model. *FGFR3* mutations might also affect membranous ossification. Gain-of-function mutations of *FGFR3* (P250R and A391E) have been shown to cause human Muenke syndrome and Crouzon syndrome with acanthosis nigricans^20^,^21^. Affected patients undergo premature fusion of the cranial sutures. Furthermore, TD patients also frequently exhibit severe craniosynostosis phenotypes^22^. ACH patients present craniofacial phenotypes suggesting the possibility of craniosynostosis defects. Recent report showed that ACH is associated with craniosynostosis and suggested that craniosynostosis may be under-reported^23^. An increased understanding of premature fusion of the cranial sutures and craniofacial phenotypes in ACH at early stages will facilitate improved treatment of these defects. For the first time, we have demonstrated here that the cranial sutures undergo premature fusion in newborn ACH mice, which offers the opportunity to better understand the development of cranial anomalies in ACH, and further study membranous ossification.

During the postnatal and adult stages, bone undergoes continuous remodelling through the coordinated processes of bone formation and bone resorption^24^. Low bone density has been reported in adult ACH^19^, which may have clinical relevance and lead to subsequent bone damage, such as increased fragility and risk of fracture. Enhanced osteoblast differentiation has been observed in long bone growth plates of *Fgfr3*^*G369C*^ mice at the age of 15 days^9^, suggesting advanced ossification at an early stage. Recent studies in *Fgfr3*^*G369C*^ mice revealed enhanced osteogenic differentiation in cultured bone marrow stromal cells, and this was associated with decreased bone mass at 2 months of age^25^. Here, we demonstrated that the expression of osteocalcin was increased in the chondrocytes of the hypertrophic zone of the distal femur in *FGFR3*^*ACH*^ mice at 2 weeks of age, suggesting enhanced osteoblast differentiation in *FGFR3*^*ACH*^ mice. Recently, a study revealed that bone density was reduced in the majority of ACH and HCH patients in the age range of 10–33 years, which is indicative of osteopenia^26^. Here, we provide direct evidence showing a low bone density in newborn and adult *FGFR3*^*ACH*^ mice. Furthermore, we observed fewer osteoclasts and osteoblasts in the femur of *FGFR3*^*ACH*^ mice at 1 year of age. Both endochondral ossification and bone remodelling regulate bone mass in adults, suggesting that altered bone remodelling might also contribute to the lower bone mass of adult *FGFR3*^*ACH*^ mice. Potential changes in bone structure should be further evaluated in neonatal ACH patients to determine whether an adequate diet and exercise may help to prevent any such osteopenia at early developmental stages. Our mouse model offers a good opportunity for testing interventions for early onset osteopenia in ACH.

The mouse is a convenient animal model for the study of human genes and diseases, which has led to the development of treatments for many serious diseases and conditions. However, mice are not always reliable as preclinical models for human disease. Many drugs have shown promising results in preclinical trials in mice but later failed in human clinical trials. As such, there is a need to develop reliable preclinical mouse models of human diseases for clinical research and drug development. Mice expressing a mutated version of a human gene known to be associated with a specific human disease and faithfully mimicking the disease phenotypes can be useful for studying disease pathology, conducting preclinical research, and testing compound efficacy *in vivo*. We have generated an ACH mouse model that faithfully and comprehensively recapitulates the disease phenotypes, enabling the evaluation of potential treatments targeting the over-activation of human *FGFR3* and its downstream signaling.

## Methods

(Full experimental details are provided in the Supplemental Data).

### Mice

Mice were housed in a temperature- and humidity-controlled room with a 12-h light/12-h dark cycle under specific pathogen-free conditions. All animal protocols were approved by the Institutional Animal Care and Utilization Committees, Academia Sinica, Taiwan (Protocol #14-12-795). The investigation conformed to the Guide for the Care and Use of Laboratory Animals published by the US National Institutes of Health.

### Gene targeting and generation of chimeric mice

To generate a targeting vector for the expression of human *FGFR3*^G380R^, we adopted a highly efficient recombination-based method, as previously described^27^. The human mutant *FGFR3* cDNA (NM_000142) encoding the *FGFR3*^G380R^ protein was used to replace the WT allele of *Fgfr3* in 129 Sv mice. The stem cells carrying the targeting vector without the neomycin resistance cassette were injected into C57BL/6 J blastocysts^28^. The resulting chimeric mice were crossed with 129 Sv females to enable germline transmission. Heterozygotes were used to continue the strain and to provide experimental pairs.

### Quantitative reverse transcriptase polymerase chain reaction (qRT-PCR)

Total RNA from the left hind-limb of neonatal mice was isolated, converted to cDNA, and subjected to qRT-PCR. Expression data were normalized to Gapdh mRNA levels. Gene-specific primer sequences are listed below.

Mouse *Fgfr3*: forward sequences, 5′-TGCGGTGCCTTCACAGA-3′; reverse sequences, 5′-ACTTGGACCTCTCCGTG-3′.

Human *FGFR3*: forward sequences, 5′-GCTGAGGACACAGGTGTG-3′ ; reverse sequences, 5′-CACTCCCTCCATCTCCTG-3′.

Gapdh: forward sequences, 5′-CCAGAACATCATCCCTGCAT-3′; reverse sequences, 5′-GTTCAGCTCTGGGATGACCTT-3′.

### Bone length measurement

Femurs of mice were dissected and the flesh was removed. The lengths of the femurs were measured using a millimetre-scale calliper ruler.

### Micro-CT

Two-dimensional imaging of whole mice, skulls, shoulder joints, and hind limbs was performed on euthanised mice using a Skyscan 1076 system (Bruker, Brussels, Belgium). The distal femur metaphyses of fixed trabecular bones were analysed by three-dimensional micro-CT using a Skyscan 1076 3D system in the Taiwan Mouse Clinic, following their standard protocol (as described in a previous report)^29^. The following scanning parameters were chosen: image pixel size: 9 μm, X-ray voltage: 50 kV, X-ray current: 140 μA, filter: A1 0.5 mm, exposure: 3300 ms, rotation step: 0.8°, frame averaging: 2, tomographic rotation: 180°. Cross-sections were reconstructed using NRecon software (Bruker). The parameters were as follows: smoothing: 0, ring artefacts reduction: 6, beam-hardening correction: 20%, change dynamic image range: 0.015–0.07.

### Histology, histochemistry, and immunohistochemistry

Sections of fixed bone tissues were prepared and examined with Masson’s trichrome stain. For immunohistochemistry, the sections were incubated with phospho-*FGFR3* antibody (Cell Signaling, Danvers, MA, USA) or anti-osteocalcin antibody (Millipore, Billerica, MA, USA) and then visualised using 3,3′-diaminobenzidine.

### Primary chondrocyte culture and cell proliferation assay

The primary chondrocytes were isolated and cultured as previously described^30^ with several modifications as described in the Supplementary Data. The proliferation of primary chondrocytes (we did not use subcultured cells) was assessed using an iCELLigence™ real-time cell analyser (Acea Biosciences, San Diego, CA, USA, distributed by Roche Diagnostics, Basel, Switzerland). The changes in adhesion and spreading of the cells were continuously recorded for 15 days using the iCELLigence™ system. Data were expressed as a graph of cell index values during the exponential phase.

### Skeletal preparation

Skinned and eviscerated newborn mice were fixed and stained with Alcian blue 8GX (Sigma–Aldrich, St. Louis, MO, USA) and alizarin red (Sigma–Aldrich).

### Statistical analysis

A two-tailed Student’s *t*-test was used to test for differences between groups. A *p* value less than 0.05 was considered to be statistically significant (*p < 0.05, **p < 0.01, ***p < 0.001). Chi-square goodness-of-fit tests (2 degrees of freedom) were used to test for departures from Mendelian expectations for the genotypes of *FGFR3*^*ACH*^mice generated from heterozygous breeding pairs.

## Additional Information

**How to cite this article**: Lee, Y.-C. *et al*. Knock-in human *FGFR3* achondroplasia mutation as a mouse model for human skeletal dysplasia. *Sci. Rep.* **7**, 43220; doi: 10.1038/srep43220 (2017).

**Publisher's note:** Springer Nature remains neutral with regard to jurisdictional claims in published maps and institutional affiliations.

## Supplementary Material

Supplementary Data

## Figures and Tables

**Table 1:** Body length and femur length of 1-month-old mice.

	Body length	Femur length
	(mm, *n* = 14)	(mm, *n* = 9)
WT	72.69 ± 4.905	12.33 ± 0.5
ACH/+	67.52 ± 4.332***	9.61 ± 0.8529***
Percentage decreased	7.11%	22.06%

WT: wild-type littermates; ACH/+: *FGFR3*^*ACH*/+^ mice. Each value is expressed as the mean ± SD (*n* values shown in column headers). ***p < 0.001.

**Table 2:** Structural parameters of distal femur trabecular bone in newborn mice.

	BV/TV (%)	Tb.Th (mm)	Tb.Sp (mm)	Tb.N (1/mm)	SMI
newborn					
WT (*n* = 3)	43.16 ± 1.85	0.076 ± 0.007	0.086 ± 0.006	5.718 ± 0.35	1.487 ± 0.24
ACH/ACH (*n* = 3)	15.76 ± 3.93***	0.058 ± 0.011	0.17 ± 0.045	2.74 ± 0.71***	2.436 ± 0.26**
1 year old					
WT (*n* = 4)	5.22 ± 0.51	0.079 ± 0.010	0.383 ± 0.047	0.661 ± 0.087	2.569 ± 0.20
ACH/ACH (*n* = 3)	2.74 ± 1.08*	0.069 ± 0.003	0.378 ± 0.075	0.402 ± 0.169	2.87 ± 0.37

WT: wild-type littermates; ACH/ACH: *FGFR*^*ACH/ACH*^ mice. BV/TV: trabecular bone volume/tissue volume; Th.Th: trabecular thickness; Th.Sp: trabecular separation; Tb.N: trabecular number; SMI: structure model index. Each value is expressed as the mean ± SD (all groups *n* = 3). *p < 0.05; ***p < 0.01; ***p < 0.001.

**Table 3:** Similarity of skeletal features found in human achondroplasia and observed in achondroplasia mouse models.

Skeletal features of human achondroplasia	Tg m*Fgfr*^*ACH*^	KI m*Fgfr3*^*ACH*^	Tg h*FGFR3*^*ACH*^	KI h*FGFR3*^*ACH*^
Rhizomelic dwarfism at birth	NS	NS	At birth^a^	At birth
Large head with frontal bossing, mid-face hypoplasia at birth	21 days	10 days	At birth	At birth
Craniosynostosis at birth	ND	ND	ND	At birth
Low bone density in adolescent and adult	ND	ND	ND	At birth
Homozygous ACH patients are stillborn or die during the neonatal period	ND	ND	Die soon after birth	A higher mortality rate at birth
Thoracic kyphosis by 4 months	1 month	5 weeks	ND	1 month
Narrow growth plate	10 days	1 month	At birth	1 month

**Tg m*****Fgfr3***^***ACH***^, transgenic mice expressing mouse *Fgfr3*^G374R^ using the type II collagen promoter and enhancer sequences^10^. **KI m*****FGFR3***^***ACH***^, gene targeting mouse *Fgfr3*^G374R^ 8. **Tg h*****FGFR3***^***ACH***^, transgenic mice expressing human *FGFR3*^G380R^ using the mouse *Fgfr3* promoter^11^. **KI h*****FGFR3***^***ACH***^, gene targeting human *FGFR3*^G380R^ as described in this report. **ND,** not described; **NS**, not significant.

^a^The time point when the specific phenotype was first observed in each ACH mouse model.
