# Supplementary material for: Knock-in human FGFR3 achondroplasia mutation as a mouse model for human skeletal dysplasia
Source: Sci Rep. 2017 Feb 23;7:43220. doi: 10.1038/srep43220 (PMC5322349; doi:10.1038/srep43220)
Supplement: Supplementary Data [file srep43220-s1.pdf]

Knock-in human *FGFR3* achondroplasia mutation as a mouse model for human skeletal dysplasia

Yi-Ching Lee[1]*, I-Wen Song[2], Ya-Ju Pai[2]†, Sheng-De Chen[2], Yuan-Tsong Chen[2, 3]*

[1]Institute of Cellular and Organismic Biology, Academia Sinica, Taipei, 11529, Taiwan.

[2]Institute of Biomedical Sciences, Academia Sinica, Taipei, 11529, Taiwan.

[3]Department of Pediatrics, Duke University Medical Center, Durham, NC, 27710, USA.

Supplemental Data

Supplemental Materials and Methods

Gene targeting and generation of chimeric mice

To generate a targeting vector for the expression of human FGFR3^{G380R}, we adopted a highly efficient recombination-based method, as previously described[1]. We used multi-step recombinant engineering to generate multiple vectors through a series of recombination steps. A DNA fragment carrying the entire mouse *Fgfr3* locus derived from the 129S7 mouse strain was retrieved from a bacterial artificial chromosome clone (Geneservice, Cambridge, UK) and cloned into the PL253 plasmid (Fig 1A. I). A *loxP*-flanked neomycin resistance cassette was inserted into exon 18 after the stop codon through recombination, and the neomycin resistance cassette was removed through Cre/*loxP* excision, leaving behind a *loxP* sequence (Fig 1A. II). The human mutant *FGFR3* cDNA (NM_000142) encoding the FGFR3^{G380R} protein introduced three translational stop codons followed by a neomycin resistance cassette flanked by a *FRT* site, and a *loxP* sequence was inserted after the mouse exon 2 start codon. The region of mouse *Fgfr3* between exons 2–18 was removed by Cre/*loxP* excision (Fig 1A. III). The PL253 targeting vector containing the human mutant *FGFR3* cDNA with three stop codons followed by a *FRT*-flanked neomycin resistance cassette was used to replace the WT allele of *Fgfr3* in 129Sv mouse embryonic stem cells (Fig. 1A. IV). The final structures of the targeting vector construct with and without the

neomycin resistance cassette are also illustrated. Clones of these cells containing the targeted allele were identified by Southern blot analysis (Fig. 1B) through BglI digestion and probing with a 0.5 kb 3′ oligonucleotide probe (Fig. 1A) generated by PCR. Southern blot analysis of BglI-digested DNA isolated from WT and carrier embryonic stem cells showing the validation of the targeting event by the presence of the BglI 8.4 kb fragment. The neomycin resistance cassette in the identified stem cells was removed by Flp/*FRT* excision, and the cells were then analysed by PCR with primers against the end of human *FGFR3* cDNA and exon 18 of mouse *Fgfr3* (see Fig. 1A), followed by EcoRI digestion of the PCR products (Fig. 1C). The stem cells carrying the targeting vector without the neomycin resistance cassette were injected into C57BL/6J blastocysts[2]. The resulting chimeric mice were crossed with 129Sv females to enable germline transmission. Heterozygotes were used to continue the strain and to provide experimental pairs.

Micro-computed tomography (micro-CT)

Two-dimensional imaging of whole mice, skulls, shoulder joints, and hind limbs was performed on euthanised mice using a Skyscan 1076 system (Bruker, Brussels, Belgium). Mice were then sacrificed and the dissected tissues were fixed in 4% (w/v) paraformaldehyde overnight. After transferring the specimens to 70% (v/v) ethanol, we analysed the trabecular bone of the distal femur metaphysis by three-dimensional micro-CT using a Skyscan 1076 3D system in the Taiwan Mouse Clinic, following their standard protocol (as described in a previous report)[3]. The following scanning parameters were chosen: image pixel size: 9 μm, X-ray voltage: 50 kV, X-ray current: 140 μA, filter: A1 0.5 mm, exposure: 3300 ms, rotation step: 0.8°, frame averaging: 2, tomographic rotation: 180°. Cross-sections were reconstructed using NRecon software (Bruker). The parameters were as follows: smoothing: 0, ring artefacts reduction: 6, beam-hardening correction: 20%, change dynamic image range: 0.015–0.07.

Histology, histochemistry, and immunohistochemistry

Bone tissues were fixed with 4% (w/v) paraformaldehyde, and 5 μm sections were prepared and examined with Masson's trichrome stain. For immunohistochemistry, the fixed bone tissues were then decalcified in 10% (w/v) EDTA for 2 weeks. The sections were de-paraffinised and retrieved by incubation in 0.05% (w/v) trypsin at 37 °C for 15 min. After treatment with 3% (v/v) H_2O_2 and blocking with 5% (v/v) normal goat serum, sections were incubated with primary antibodies at 4 °C overnight. Phospho-FGFR3 antibody (Cell Signaling, Danvers, MA, USA) was used to detect FGFR3 activation, and the sections were then incubated with anti-rabbit secondary antibody (VECTASTAIN® ABC system, Vector Labs, Burlingame, CA, USA) and visualised using 0.1% (w/v) 3,3′-diaminobenzidine. Images were captured

using standard light microscopy (Zeiss, Oberkochen, Germany).

Primary chondrocyte culture

The primary chondrocytes were isolated and cultured as previously described[4] with several modifications. Briefly, the connective tissue-free epiphysis region was dissected from day 10 postnatal mice and then digested with type II collagenase (Worthington, Lakewood, NJ, USA) overnight. Chondrocytes were filtered through a 70-mm nylon mesh (BD Biosciences, San Jose, CA, USA) and then cultured in DMEM containing 10% (v/v) FCS. To avoid transformation of the cell phenotype, we only used primary cells for experiments (i.e., we did not use subcultured cells).

Cell proliferation assay

Chondrocyte proliferation was assessed using an iCELLigence™ real-time cell analyser (Acea Biosciences, San Diego, CA, USA, distributed by Roche Diagnostics, Basel, Switzerland) [35]. Briefly, 10,000 viable cells/well were seeded in an 8-well plate in complete medium and grown for 10 days. The changes in adhesion and spreading of the cells were continuously recorded for 15 days using the iCELLigence™ system. The growth medium was exchanged every 2 days. Data were expressed as a graph of cell index values during the exponential phase.

Skeletal preparation

Skinned and eviscerated newborn mice were fixed overnight in 95% (v/v) ethanol, followed by overnight incubation with acetone. The specimens were then stained with Alcian blue 8GX (0.05%, w/v) (Sigma–Aldrich, St. Louis, MO, USA) for 72 h, after which they were dehydrated in 95% (v/v) ethanol for 24 h. The skeletons were incubated in 1% (w/v) KOH until the bone was visible. The skeletons then were further stained with alizarin red (0.005%, w/v) (Sigma–Aldrich) for 24 h. Specimens were cleared in a solution of 35% ethanol, 50% glycerol, and 15% water (all percentages represent v/v) to remove excess stain, and then preserved in 100% glycerol.

Supplemental Figures

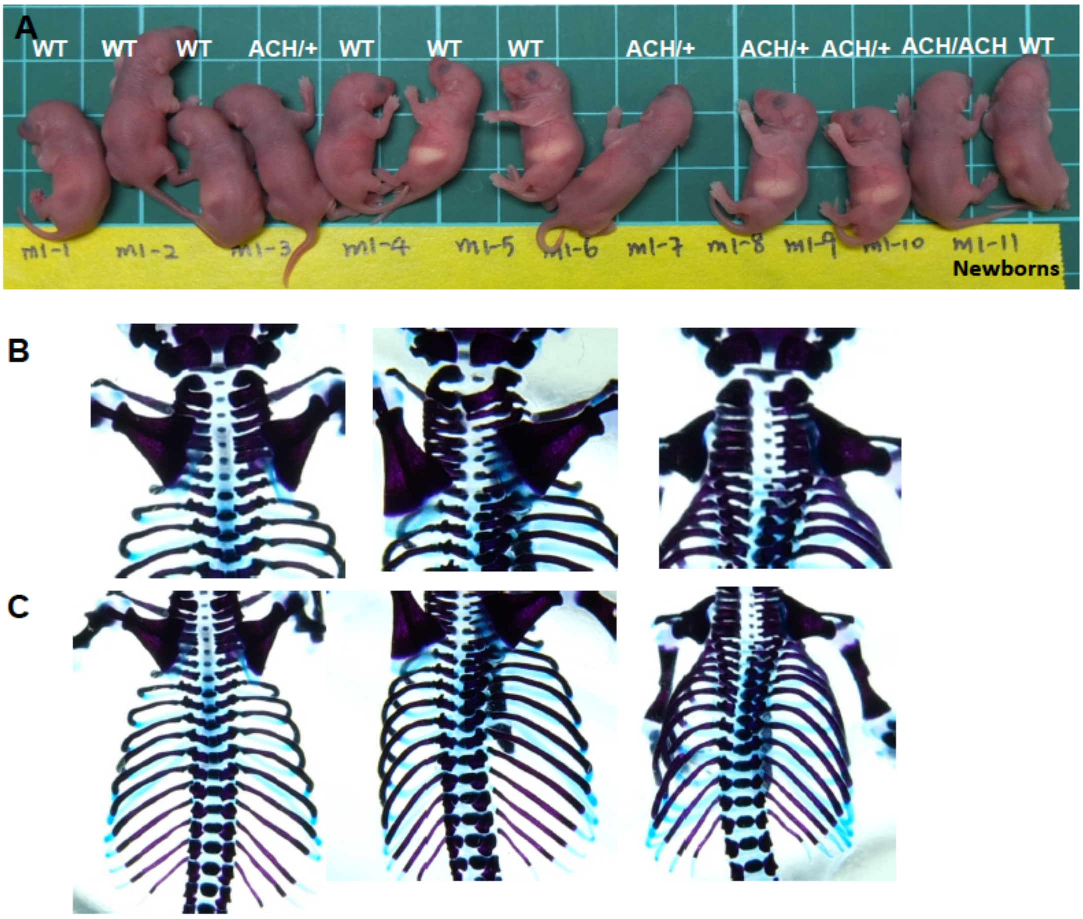

Supplemental Figure 1. Skeletal defects and changes in bone architecture in newborn *FGFR3*^{ACH} mice. (A) There were no obvious differences in appearance among the bones of the *FGFR3*$^{ACH/+}$, *FGFR3*$^{ACH/ACH}$, and WT neonates. (B) Dorsal view for comparison of the cervico-thoracic spine. (C) Dorsal view for comparison of the rib cages.

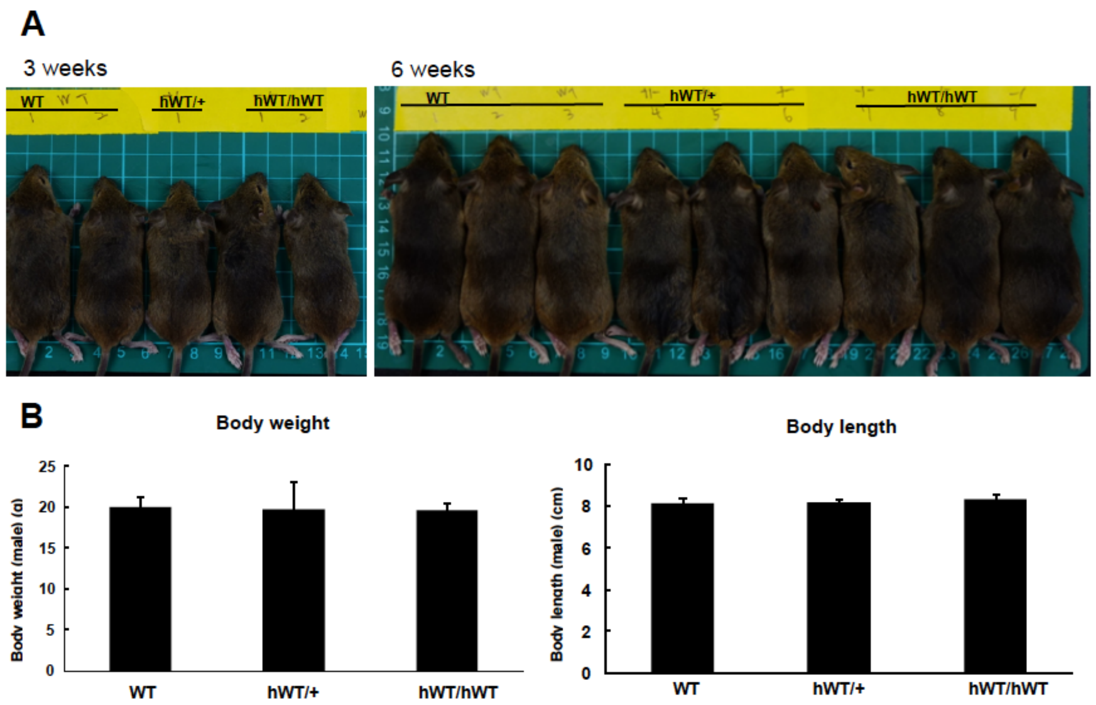

Supplemental Figure 2: The phenotypes of *FGFR3*^{WT} mice. (A) There were no obvious differences in appearance among *FGFR3*$^{WT/+}$, *FGFR3*$^{WT/WT}$, and WT mice at 3 and 6 weeks of age. (B) There were no differences in the body weight or body lengths of *FGFR3*$^{WT/+}$, *FGFR3*$^{WT/WT}$, and WT mice at 1 month of age. n = 6 for each group. Each column shows the average body weight or body length of male animals from several litters and represents the mean value ± SD.

Supplemental References

1 Liu, P., Jenkins, N. A. & Copeland, N. G. A highly efficient recombineering-based method for generating conditional knockout mutations. *Genome Res* **13**, 476-484, doi:10.1101/gr.749203 (2003).

2 Yu, I. S. *et al.* TXAS-deleted mice exhibit normal thrombopoiesis, defective hemostasis, and resistance to arachidonate-induced death. *Blood* **104**, 135-142, doi:10.1182/blood-2003-10-3661 (2004).

3 Saleem, A. N. *et al.* Mice with alopecia, osteoporosis, and systemic amyloidosis due to mutation in Zdhhc13, a gene coding for palmitoyl acyltransferase. *PLoS genetics* **6**, e1000985, doi:10.1371/journal.pgen.1000985 (2010).

4 Gosset, M., Berenbaum, F., Thirion, S. & Jacques, C. Primary culture and phenotyping of murine chondrocytes. *Nature protocols* **3**, 1253-1260, doi:10.1038/nprot.2008.95 (2008).
